# Percutaneous transthoracic coil embolization of a gutter-related type Ia endoleak after chimney thoracic endovascular aortic repair

**DOI:** 10.1016/j.jvscit.2025.101906

**Published:** 2025-07-07

**Authors:** Emiel W.M. Huistra, Ignace F.J. Tielliu, Aryan Mazuri, G. Matthijs Kater, Clark J. Zeebregts

**Affiliations:** a Division of Vascular Surgery, Department of Surgery, University Medical Center Groningen, University of Groningen, Groningen, the Netherlands; b Department of Radiology, University Medical Center Groningen, University of Groningen, Groningen, the Netherlands

**Keywords:** Chimney graft, Thoracic endovascular aortic repair, Gutter endoleak, Type I endoleak, Left subclavian artery, Coil embolization

## Abstract

This report describes the treatment of a gutter-related type Ia endoleak after chimney thoracic endovascular aortic repair to the left subclavian artery. The aneurysm sac was accessed via direct percutaneous transthoracic puncture, followed by selective catheterization of the gutter. Angiography confirmed the endoleak, which was treated with coil embolization. Track embolization was performed using Histoacryl glue. Follow-up computed tomography angiography demonstrated complete resolution of the endoleak. Percutaneous transthoracic coil embolization seems to be a feasible and effective treatment for gutter-related type Ia endoleak after chimney thoracic endovascular aortic repair. Given the anatomical complexity and variation in access options, treatment strategies should be individualized.

Thoracic endovascular aortic repair (TEVAR) is a minimally invasive alternative to open surgery for treating pathologies of the descending thoracic aorta.^1^ To ensure adequate seal of the endograft, the left subclavian artery (LSA) is frequently overstented, either with or without revascularization.^2^ Preserving blood flow to the LSA potentially decreases neurological complications, including stroke, spinal cord ischemia, and arm ischemia.^3^ The optimal method for LSA revascularization remains a topic of debate.^4^

In chimney TEVAR, parallel stents are used to maintain perfusion of the aortic side branches. This approach offers an acceptable technical success rate and good mid-term patency of chimney stents.^5^^,^^6^ However, chimney TEVAR is associated with a relatively high incidence of gutter-related type Ia endoleaks.^7^^,^^8^ This report describes a percutaneous transthoracic approach to coil embolization of a gutter-related type Ia endoleak after chimney TEVAR. Written informed consent for publication was obtained from the patient.

## Technique

**Fig 1:**
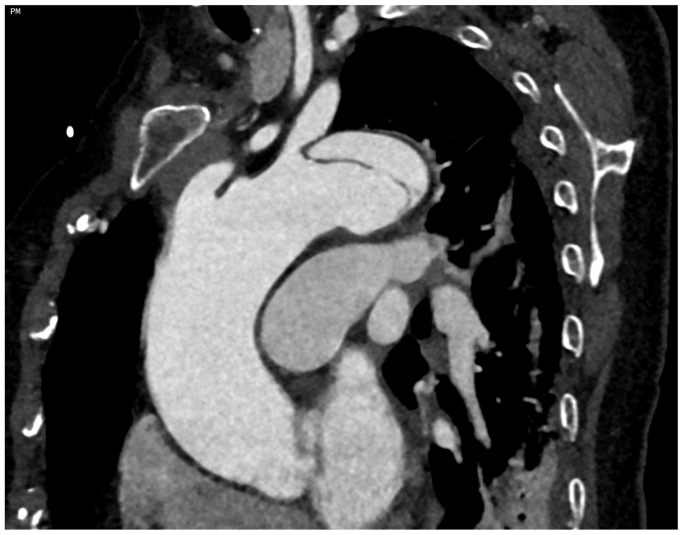
Computed tomography angiography (CTA) showing the type B dissection before endovascular treatment, with the location of the primary entry tear distal to the left subclavian artery (LSA).

**Fig 2:**
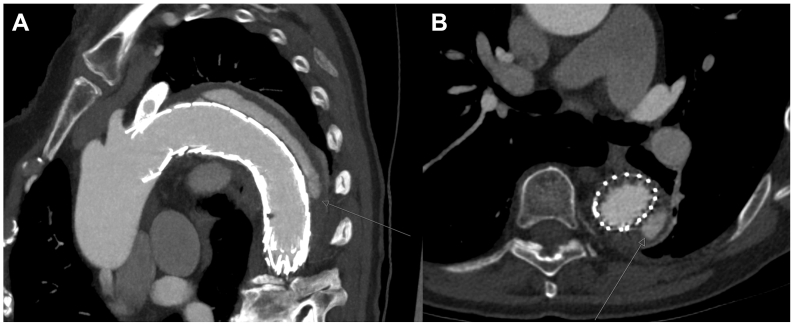
**(A, B)** Computed tomography angiography (CTA) demonstrating a suspected gutter-related type Ia endoleak after left subclavian artery (LSA) chimney thoracic endovascular aortic repair (TEVAR) for a complicated type B aortic dissection. The *arrow* indicates the percutaneous transthoracic access route to the false lumen.

This technique was applied in an 82-year-old patient with a history of hypertension and an acute type B dissection, complicated by uncontrolled hypertension and refractory pain (Fig 1). The type B dissection had been treated previously with a 40 × 200 mm TEVAR (TAG Conformable Thoracic Stent Graft; W. L. Gore & Associates, Flagstaff, AZ), achieving approximately 18 % oversizing in a 34-mm-wide landing zone, and a 11 × 59 mm chimney stent (Viabahn VBX Balloon Expandable Endoprosthesis; W. L. Gore & Associates) for the LSA. The chimney stent overlapped with the TEVAR graft for approximately 15.5 mm. Computed tomography angiography (CTA) 5 days postoperatively revealed a small endoleak. At the 1-month follow-up, CTA demonstrated a significant type Ia endoleak, presumed to originate from the gutter, along with a 4-mm increase in the aortic diameter (Fig 2, *A* and *B*). Coil embolization of the endoleak via a transthoracic approach was planned subsequently.

The procedure was performed in a hybrid operating room with the patient under general anesthesia in the prone position. Using biplane fluoroscopic guidance, the aneurysm sac was directly punctured transcutaneously at the level of the seventh thoracic vertebra using an 18G, 15-cm needle. After successful access to the aneurysm sac, a 6F predilatation was performed over an Amplatz guidewire (Cook Medical, Bloomington, IN), followed by the introduction of a 4F, 55-cm sheath.

**Fig 3:**
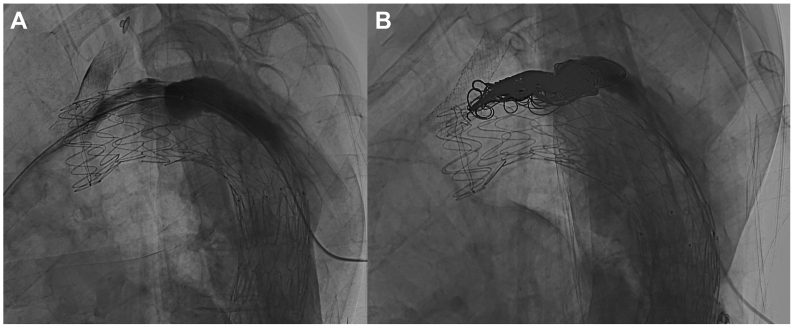
**(A)** Digital subtraction angiography after transthoracic access to the false lumen showing a type Ia endoleak and **(B)** subsequent resolution of the type Ia endoleak after coil embolization of the gutter.

Access to the ascending aorta was obtained via the gutter between the chimney stent and the main endograft. Initial digital subtraction angiography of the aortic arch and distal TEVAR segment revealed no evidence of an endoleak. However, contrast injection into the false lumen showed a type Ia endoleak (Fig 3, *A* and *B*). These seemingly contradictory findings may be explained by the lack of outflow through collateral arteries, resulting in an elevated pressure within the false lumen, impeding antegrade contrast flow. These observations were consistent with the preoperative CTA, which demonstrated a visible endoleak with minimal contrast in the gutter and no apparent inflow or outflow via collateral arteries.

**Fig 4:**
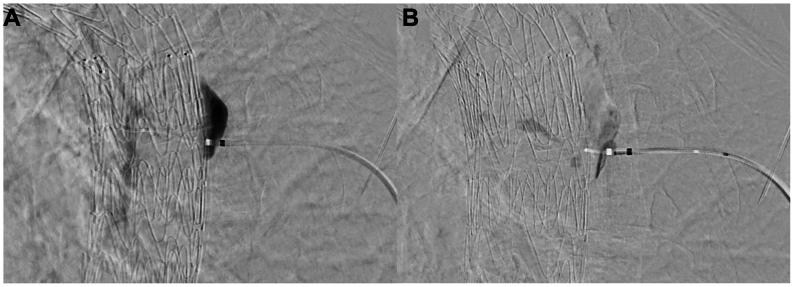
**(A)** Retraction of the sheath over a microcatheter while injecting contrast to delineate the outer edge of the aorta, identified by a change in the pattern of contrast dispersion, and **(B)** subsequent glue embolization through the microcatheter, beginning within the false lumen and extending to just beyond the outer edge of the aortic wall.

**Fig 5:**
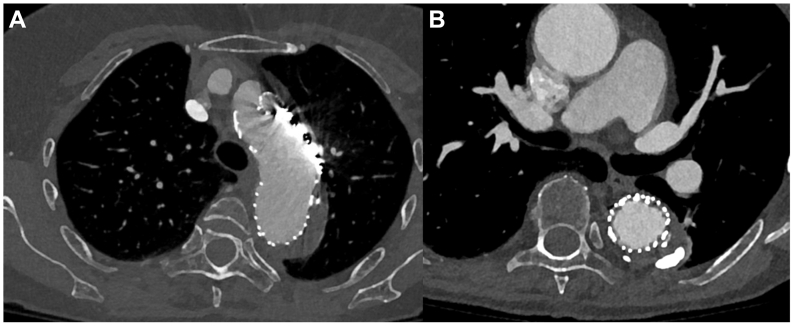
**(A)** Computed tomography angiography (CTA) 2 months after percutaneous transthoracic retrograde coil embolization of a gutter endoleak demonstrating complete resolution of the endoleak and **(B)** the presence of Histoacryl glue at the level of the puncture site.

Coil embolization was performed using Interlock (Boston Scientific, Marlborough, MA) and Prestige (Balt Extrusion, Montmorency, France) coil systems. A total of 769 cm of coiling material was deployed. Track embolization was completed with Histoacryl glue (B. Braun, Melsungen, Germany) during sheath withdrawal (Fig 4, *A* and *B*). The fluoroscopy time was 72 minutes, and the dose area product was 207 Gy·cm^2^. The patient's recovery was uneventful, and follow-up CTA at 2 months confirmed complete resolution of the endoleak (Fig 5, *A* and *B*).

## Discussion

Chimney stents provide an effective solution when TEVAR necessitates coverage of a supra-aortic branch. However, type Ia endoleak owing to gutter formation remains a concern.^5^, ^6^, ^7^, ^8^ This report demonstrates the feasibility of a percutaneous transthoracic approach for treating such endoleaks.

A meta-analysis by Hogendoorn et al^5^ reported a 6.4% incidence of type Ia endoleaks, with 50% requiring reintervention. In contrast, Ullery et al^9^ found that, among 60 patients who underwent chimney EVAR for complex abdominal aortic aneurysm, a type Ia gutter endoleak was identified in 30% on early postoperative imaging, with only 3.3% requiring secondary intervention. Notably, no significant difference in aneurysm sac size change was observed between patients with or without early type Ia gutter endoleak.

The variability in reported incidence rates and the uncertain natural course of gutter-related type Ia endoleaks highlight the challenges in establishing a standardized treatment. Ballesteros-Pomar et al^10^ proposed an algorithm for managing type Ia gutter endoleaks after chimney EVAR for abdominal aortic aneurysms, which may offer generalizable principles applicable to thoracic cases. Their approach includes intraoperative kissing balloon dilatation of the aortic stent grafts and chimney grafts, ensuring an adequate sealing zone length and appropriate oversizing. Follow-up imaging is performed at 3 months, and persistent endoleaks are treated with coils or liquid embolic agents. Open surgical conversion, which in the context of TEVAR may require a frozen elephant trunk procedure, is reserved for persistent endoleaks that cause significant enlargement of the aneurysm sac despite embolization.

Alternative treatment methods have also been proposed, such as the use of Aptus Heli-FX EndoAnchors (Medtronic Cardiovascular, Santa Rosa, CA), or limiting reinterventions to patients with recurrent symptoms or aneurysm sac expansion of more than 5 mm.^9^^,^^11^ Prophylactic coil embolization at the time of initial TEVAR has also been suggested for pronounced gutter-related endoleaks.^12^

Marcelin et al^13^ demonstrated the feasibility and safety of gutter-related endoleak embolization in a series of nine patients, achieving 100% technical success and high clinical efficacy. However, these procedures were performed after abdominal EVAR, which allows for antegrade access to the gutter endoleak. In contrast, accessing a type Ia gutter endoleak in the aortic arch is challenging because of the unfavorable catheter angles required via a femoral or aortic branch approach. Moreover, excessive catheter manipulation in the aortic arch may increase the risk of cerebral embolization and stroke. Nonetheless, successful coiling of type Ia endoleaks in the aortic arch via femoral access has been reported.^14^^,^^15^ Other access routes, such as a trans-septal approach, have been suggested to allow straight-line access to the gutter between the endograft and the aortic wall.^16^

Retrograde embolization of a gutter through the false lumen has been described as an alternative technique after chimney TEVAR, using the transfemoral route to reach the false lumen through a distal reentry tear.^17^ The direct percutaneous transthoracic retrograde coil embolization described in the current report may be applicable, even in patients lacking favorable anatomy for transfemoral false lumen access.

Two previous reports have described successful transthoracic percutaneous coil embolization for type Ia endoleak. Katada et al^18^ performed the procedure with the patient in the prone position to treat a type Ia endoleak after standard TEVAR, and Bangard et al^19^ used a parasternal approach with the patient in the supine position to treat a persistent type Ia endoleak after TEVAR despite proximal extension with a double chimney technique. The current report further supports the feasibility of percutaneous transthoracic coil embolization as a primary treatment strategy for type Ia gutter endoleak after chimney TEVAR. A potential risk is pneumothorax, and preoperative planning should include an assessment of the pleural anatomy to ensure a safe puncture route.

Because of the anatomical variability and limited comparative data across available access routes, treatment strategies should be individualized, with access route selection based on patient anatomy, location of the endoleak, prior treatment history, and institutional experience. In this case, percutaneous transthoracic retrograde embolization was considered the safest and most technically feasible option. In particular, it allowed for improved control over catheter positioning and facilitated achieving dense and effective coil packing within the gutter. Attempting this maneuver from an antegrade approach via the femoral approach would have introduced technical challenges, particularly with regard to catheter stability and coil density, and would have required more extensive manipulation near the supra-aortic branches, which was avoided to minimize the risk of an embolic event.

Although glue embolization can be considered, it was avoided in the current case owing to the risk of distal migration and stroke. Coil migration is very unlikely, and, in this case, the exclusive use of coils resulted in complete obliteration of the endoleak. Owing to the presence of embolic material and limited follow-up duration, detecting persistent slow-flow endoleaks remains challenging. Longer-term follow-up is necessary to confirm durability.

## Conclusions

Percutaneous transthoracic retrograde coil embolization is a feasible and effective treatment for gutter-related type Ia endoleak after chimney TEVAR. Longer-term follow-up is needed to confirm the durability of this approach. Because of anatomical variability and limited comparative data across available access routes, treatment strategies should be individualized, with access route selection based on patient anatomy and institutional experience.

## Funding

None.

## Disclosures

C.Z. is a consultant for and has received research support, honoraria, and travel support from W. L. Gore & Associates, LeMaitre Vascular, Atrium Maquet Getinge Group, Artivion, Terumo (Vascutek and Bolton), and Cook Medical.
